# Perceived stigma among discharged patients of COVID-19 in Wuhan, China: A latent profile analysis

**DOI:** 10.3389/fpubh.2023.1111900

**Published:** 2023-03-21

**Authors:** Yijin Wu, Zhenwei Dai, Weijun Xiao, Hao Wang, Yiman Huang, Mingyu Si, Jiaqi Fu, Xu Chen, Mengmeng Jia, Zhiwei Leng, Dan Cui, Winnie W. S. Mak, Xiaoyou Su

**Affiliations:** ^1^School of Population Medicine and Public Health, Chinese Academy of Medical Sciences & Peking Union Medical College, Beijing, China; ^2^National Clinical Research Center for Respiratory Diseases, China-Japan Friendship Hospital, Beijing, China; ^3^The 2nd Affiliated Hospital of Harbin Medical University, Harbin Medical University, Harbin, China; ^4^Department of Psychology, Diversity and Well-Being Laboratory, The Chinese University of Hong Kong, Shatin, Hong Kong, China

**Keywords:** COVID-19, perceived stigma, latent profile analysis, discharged patients, China

## Abstract

**Background:**

Perceived stigma has greatly influenced the life quality of the COVID-19 patients who recovered and were discharged (RD hereafter). It is essential to understand COVID-19 stigma of RD and its related risk factors. The current study aims to identify the characteristics of perceived COVID-19 stigma in RD using latent profile analysis (LPA), to explore its psycho-social influencing factors, and to determine the cut-off point of the stigma scale using receiver operating characteristic (ROC) analysis.

**Methods:**

A cross-sectional study was conducted among COVID-19 RD in 13 communities in Jianghan District, Wuhan City, Hubei Province, China from June 10 to July 25, 2021, enrolling total 1,297 participants. Data were collected on demographic characteristics, COVID-19 perceived stigma, post-traumatic stress disorder (PTSD), anxiety, depression, sleep disorder, fatigue, resilience, social support, and peace of mind. LPA was performed to identify different profiles of perceived COVID-19 stigma level. Univariate analysis and multinominal logistic regression analysis were conducted to explore the influencing factors in different profiles. ROC analyses was carried out to identify the cut-off value of perceived stigma.

**Results:**

Among the participants, three profiles of perceived stigma were identified: “low perceived COVID-19 stigma” (12.8%), “moderate perceived COVID-19 stigma” (51.1%), and “severe perceived COVID-19 stigma” (36.1%). Multinominal logistic regression analysis revealed that older age, living with other people, anxiety, and sleep disorder were positively associated with moderate perceived COVID-19 stigma, while higher educational level was negatively associated with moderate perceived COVID-19 stigma. Female, older age, living with other people, anxiety, and sleep disorder were positively associated with severe perceived COVID-19 stigma, while higher educational level, social support, and peace of mind were negatively associated with severe perceived COVID-19 stigma. ROC curve of the Short Version of COVID-19 Stigma Scale (CSS-S) for screening perceived COVID-19 stigma showed that the optimal cut-off value was ≥ 20.

**Conclusion:**

The study focuses on the issue of perceived COVID-19 stigma and its psycho-socio influencing factors. It provides evidence for implementing relevant psychological interventions to COVID-19 RD.

## 1. Introduction

COVID-19 has emerged as a global health emergency and posed a great threat to almost all countries and regions (1). It affects all segments of the population, especially the patients of COVID-19 (2). The impact is far beyond merely physical concerns. Previous studies have shown that the pandemic has led to psychological problems among patients, healthcare workers, and other caregivers (3, 4). Patients infected with COVID-19 not only suffered from illness, but also had mental health problems due to viral infection and worries about after-effects (5). Perceived stigma is prevalent among COVID-19 survivors and healthcare workers in COVID-19 designated hospitals, which has an interrelated bearing on their mental health (6, 7).

In post pandemic era, most patients of COVID-19 have been discharged (8). The mental health of those who had recovered from COVID-19 and been discharged from hospital (RD hereafter) deserve more attention during their rehabilitation (9). These patients were isolated during treatment and had limited freedom and communication with the outside world (10). Thus, their negative emotions cannot be alleviated in a short period of time. RD may have a more serious sense of loneliness and repression, as well as a higher level of psychological pressure (11). In the aftermath and the long-covid period, they may experience depression, anxiety, fatigue, post-traumatic stress disorder, and neuropsychiatric syndromes (12–14). Poor mental health condition will impact one’s social behaviors and cognitive functions. As a result, RD’s mental health should be attached much importance.

RD’s mental health condition might affect their perceived COVID-19 stigma (15). Perceived stigma is one’s personal feelings about the stressors and his projection of the feelings on others (16). From the patient’s perspective, they might feel being stigmatized if their mental health condition was poor. COVID-19 RD are at high risk of PTSD, partly because of their near death experience, delirium, and ICU-related trauma during the COVID-19 experience (17, 18). They might have uncontrollable thoughts about the experience and their image in others’ mind, which would increase their perceived stigma. Perceived stigma might also in turn predict PTSD (19). Depression is another prevalent mental issue among COVID-19 RD (20). RD with depressive symptoms might be more sensitive and pessimistic to the negative attitudes from the community, which makes them feel more stigmatized emotions (21). Besides, to contain the spread, patients are required to stay in close isolation during treatment and reduce their movement after discharge, which may lead to feelings of loneliness and fear of discrimination, thus increasing their perceived stigma (22). Peace of mind is important for them to manage stressful situations, as well as avoid the irresistible but unwanted impulses (23). Resilience is not a linear path toward happiness, but a combination of behaviors that encourages individuals and communities to persevere and move forward confronting difficult situations (24, 25). Higher level of resilience might decrease the risk of developing psychological distress, and suppress suicidal thoughts and insomnia (26, 27). Resilience might be influenced by job stress, perceived stress, and mindfulness, and be promoted by brief resilience interventions based on positive psychology (28–30). Thus, with higher level of peace of mind and resilience, patients will control their emotions better and be less sensitive to the negative attitudes from others, which might result in lower sense of perceived stigma. From the society’s perspective, low perceived social support may also lead to perceived stigma among COVID-19 RD (31). Perceived stigma might in turn increase the mental problems among RD and be detrimental to their mental health recovery (32). Therefore, the stigma among COVID-19 RD may have a certain impact on the whole population.

The perceived COVID-19 stigma in RD could be evaluated by a modified 12-item HIV stigma scale, which contains 4 sub-scales to measure personalized stigma, disclosure concerns, concerns about public attitudes, and negative self-image (33). However, this scale has no cut-off point, which makes it hard to precisely evaluate the stigma among RD. Clinical psychiatric interviews are usually regarded as the gold standard for diagnosis and the criterion for determining cut-off points of screening tools, However, the identification and diagnosis of cases with perceived COVID-19 stigma has not reached a consensus. Additionally, the characteristics and prevalence of perceived COVID-19 stigma among RD and its psycho-social influencing factors remain elusive. Currently, most previous studies focused on the recursive effect of perceived stigma on mental health without considering the possible vicious circle between mental health and perceived stigma among RD. While according to the theory of socio-ecological model, one is not a passive recipient of life events, but a key role in constructing and modifying the living system (34). It is therefore important to explore the influencing factors of perceived COVID-19 stigma among RD. The specific objectives of current study are to identify the characteristics of perceived COVID-19 stigma in RD using latent profile analysis (LPA); to explore the psycho-social influencing factors of perceived COVID-19 stigma in RD; and to determine the cut-off point of the stigma scale using ROC analysis for further evaluation and application, which may help healthcare professions and policymakers to deal with the increasing stigma and control the pandemic effectively.

## 2. Methods

### 2.1. Study design and participants

The cross-sectional study was carried out among previously-infected COVID-19 patients in Jianghan District (Wuhan, China) from June 10 to July 25, 2021. Extracted from the electronic medical records of the Jianghan District Health Bureau, a total of 3,059 COVID-19 patients met the inclusion criteria and were eligible for the study, for they were infected with the original SARS-Cov-2 strain and were diagnosed between December 10, 2019 and April 20, 2020. When they were receiving clinical re-examination, 1,601 COVID-19 survivors were invited to complete a questionnaire survey on their mental health status, and 1,541 of them who finished the survey were included in the study. All investigators and support staff in this study were trained according to the same protocol and required to have an educational background in medicine or public health. From June to July 2021, the online structured questionnaire was distributed to those who had a history of COVID-19 infection and had been discharged. All participants’ digital informed consent was obtained to ensure their voluntary participation. An online survey platform Redcap was used to disseminate the self-administered electronic questionnaires and digital consent to the target population. The study was approved by the Ethics Review Board of the Institute of Pathogen Biology, Chinese Academy of Medical Sciences (IPB-2020-22), and the Research Ethics Committee of the hospital (2021001, 20210208). The participants had to meet the following criteria: (1) over the age of 18; (2) a history of COVID-19 hospitalization; (3) proficiency in Chinese; (4) able to independently complete scale assessments with the assistance of the researchers; (5) had a mobile communication device like a smartphone and a WeChat account; (6) able to access the Internet with mobile equipment at any time; (7) had not received PTSD, depression, or anxiety interventions within 1 month before his or her enrollment in the study. Those who met any of the following criteria were excluded: (1) had serious cognitive impairment; (2) had serious heart, brain, lung, kidney, liver, and other medical diseases or tumors; (3) found it difficult to complete the questionnaire study. In total, of the 1,541 participants who completed the questionnaire, 1,297 participants were included in the final analysis based on the criteria above.

### 2.2. Measures

#### 2.2.1. Demographic characteristics

Demographic characteristics, including gender, age, region, marital status, etc., and items on COVID-19 infection, such as clinical classification of COVID-19 in patients and perceived mental health status during hospitalization, were collected.

#### 2.2.2. Stigma

The Short Version of COVID-19 Stigma Scale (CSS-S) is a 12-item scale that is employed for evaluating the perceived stigma of patients of COVID-19 during the past 2 weeks (33). The scale was reviewed by several experts in the field and was approved to use in this population. Each item is scored on a Likert scale of 1–4. Higher total scores indicate greater stigmatization. In this study, the Cronbach’s alpha of the instrument was 0.936.

#### 2.2.3. Post-traumatic stress disorder

The Impact of Events Scale-Revised (IES-R) is a 22-item scale aimed at screening posttraumatic stress symptoms in adults or older people. The items of this instrument are rated on a 5-point Likert scale from 0 to 4 (35, 36). The IES-R contains three dimensions measuring intrusion, avoidance, and hyperarousal. Respondents rate their degree of distress during the past 7 days after they have identified a specific stressful life event that occurred to them. A total score equal to or above 35 can be regarded as positive PTSD symptoms. This instrument has been proven valid and reliable among Chinese COVID-19 patients (37). In this study, the Cronbach’s alpha of the instrument was 0.965.

#### 2.2.4. Anxiety

The Generalized Anxiety Disorder Questionnaire (GAD-7) consists of 7 items that are rated on a 4-point Likert scale from 0 to 3. It was developed for measuring the severity of generalized anxiety symptoms during the past 2 weeks (38). The scores of the instrument range from 0 to 21. A cutoff score of ≥ 5 is recommended for considering significant anxiety symptoms. This instrument has demonstrated to be reliable and valid among the Chinese population (39, 40). In this study, the Cronbach’s alpha of the instrument was 0.951.

#### 2.2.5. Depression

The Patient Health Questionnaire (PHQ-9) is a 9-item questionnaire that is used for screening and monitoring depression of varying degrees of severity during the past 2 weeks (41). The items of the PHQ-9 are rated on a 4-point Likert scale ranging from 0 to 3. The total score is utilized to assess the degree of depression of participants, with scores of ≥ 5 indicating depression. This instrument has been validated among various Chinese populations (42, 43). In this study, the Cronbach’s alpha of the instrument was 0.914.

#### 2.2.6. Sleep disorder

The Pittsburgh Sleep Quality Index (PSQI) consists of 18 items and is used to measure an individual’s quality of sleep during the past 2 weeks (44). It contains seven components including subjective sleep quality, sleep latency, sleep duration, sleep efficiency, sleep disturbance, use of sleep medication, and daytime dysfunction, and each component is a 4-point Likert scaled from 0 = no difficulty to 3 = severe difficulty. The total scores range from 0 to 21 and a cutoff score of ≥ 6 is recommended for considering certain sleep disorders (45). This instrument has been validated among Chinese population (46). In this study, the Cronbach’s alpha of the instrument was 0.784.

#### 2.2.7. Fatigue

The Fatigue Scale-14 (FS-14) is a 14-item scale aiming at measuring the severity of fatigue during the past 2 weeks (47). The items of this instrument are rated on a 2-point scale of 0–1. The FS-14 contains two dimensions measuring physical fatigue and mental fatigue, respectively. Higher total scores of the 14 items indicate a higher level of fatigue. This instrument has been proved valid and reliable among Chinese (48). In this study, the Cronbach’s alpha of the instrument was 0.845.

#### 2.2.8. Resilience

The Resilience Style Questionnaire (RSQ) consists of 16 items that are rated on a 5-point Likert scaled from 1 to 5. It is used to measure the level of an individual’s resilience during the past 2 weeks (49). Higher total scores of the 16 items indicate a greater ability to recover from negative events. This instrument was developed and validated among the Chinese rural left-behind adolescents and non-local medical workers (50, 51). In this study, the Cronbach’s alpha of the instrument was 0.975.

#### 2.2.9. Social support

The level of perceived social support of the participants was measured by two items including emotional support and material support during the past 2 weeks (52). The items were: (1) “How much support can you obtain from family/friends/colleagues when you need to talk or to obtain emotional support?” and (2) “How much support can you obtain from family/friends/colleagues when you need material support (e.g., financial help)?” and each item was 11-point Likert scaled from 0 to 10. In this study, the Cronbach’s alpha of the instrument was 0.819.

#### 2.2.10. Peace of mind

The Peace of Mind Scale (PoM) comprises a total of 7 items rated on a 5-point scale ranging from 1 (“not at all”) to 5 (“all of the time”) and is used for measuring the peace of mind during the past 2 weeks (53). Higher total scores indicate a more peaceful mind. This instrument has been validated among Chinese population (53). In this study, the Cronbach’s alpha of the instrument was 0.874.

### 2.3. Statistical analysis

Descriptive analyses were performed to describe the participants’ demographic characteristics, clinical characteristics, the condition of perceived stigma, and potential influencing factors.

In the absence of an accurate and precise reference standard, LPA has been widely employed to identify the symptom characteristics and to further calculate and determine optimal cut-off points of assessment instruments (54–56). LPA is a person-centered statistical method that employs latent profile model (LPM) to divide population into multiple profiles, and it focuses on identifying latent subpopulations within a population based on a set of continuous variables (57–59). Despite the possible arbitrariness for LPA in determining the number of class members due to its semi-subjective properties, the misclassification rate is relatively low, and it could produce more reasonable results compared with some other classification approaches (60–62). Generally, in LPA, individuals assigned to the latent profile that represents the lowest level of symptoms or risks are regarded as “non-cases,” and others are considered “cases” (56). Hence, LPA was conducted to identify the characteristics of perceived COVID-19 stigma among RD. Robust maximum likelihood (MLR) estimation was employed to estimate the parameters. The Lo–Mendell–Rubin (LMR) and the bootstrap likelihood ratio test (BLRT) were performed to compare the model fit improvement between models with k classes and k-1 classes, significant *p* values indicated a better model fit with k classes. The optimal number of classes was evaluated by the entropy, Akaike Information Criterion (AIC), Bayesian Information Criterion (BIC), the adjusted Bayesian Information Criterion (aBIC), and the interpretability and definition of classifications, where an entropy value≥0.80 represented adequate quality of classification, lower AIC, BIC, and aBIC values indicate better model fit, and the “turning point” of the scree plot for the aBIC could suggest an appropriate number of classes.

After the selection of optimal model and definition of classifications, Chi-square began with the full set of demographic and clinical characteristics, PTSD, anxiety, depression, sleep disorder, fatigue, resilience, social support, and peace of mind, to evaluate their associations with different characteristics of perceived COVID-19 stigma. Statistically significant variables (*p* ≤ 0.20) in the univariate analysis were further used for stepwise multinomial logistic regression analysis. Adjusted odds ratio (AOR) and the corresponding 95% confidence intervals (95% CI) were calculated to assess the regression model results.

Receiver operating characteristic (ROC) analysis was conducted to determine the optimal cut-off value for the CSS-S. The area under the ROC curve (AUC), sensitivity, specificity, and Youden’s index value were employed to evaluate the performance of classifiers, and Youden’s index value was used to identify the optimal cut-off value. SAS9.4 and Mplus8.3 were utilized to conduct all the analyses with level of significance determined at a 0.05 value of *p*.

## 3. Results

### 3.1. Demographic characteristics

Among the 1,541 people who finished the survey questions, 1,297 questionnaires were enrolled in the data analysis. As illustrated in Table 1, over half of the participants were male (*n* = 563, 56.6%) and were less than or equal to 60 years old (*n* = 683, 52.7%). The majority of the participants were from urban areas (*n* = 1,136, 87.6%) and married (*n* = 1,105, 85.2%). Most of the participants had an income for 2020 less than 60,000 China Yuan(CNY, 1 CNY equals 0.14 USD on 2022.12.31; *n* = 805, 62.1%), and had an education level as senior high school or below (*n* = 921, 71%). A small percentage of participants lived alone (*n* = 158, 12.2%), used alcohol no less than 2 times per week (*n* = 117, 9%), and were current smokers (*n* = 161, 12.4%). The COVID-19 patients were clinically classified into four categories: asymptomatic (*n* = 60, 4.6%), mild (*n* = 927, 71.5%), moderate (*n* = 132, 10.2%), critically severe (*n* = 178, 13.7%). A significant proportion of the participants had no experience at ICU (*n* = 1,250, 96.4%), had never received psychological or emotional counseling during hospitalization (*n* = 1,225, 94.4%), and had never received psychological or emotional counseling before infection (*n* = 1,169, 90.1%). Just under a half of participants stayed over 20 days in hospital (*n* = 611, 47.1%), and had no complication (*n* = 530, 40.9%). Most of the patients perceived good (*n* = 736, 56.7%) or moderate (*n* = 247, 19%) mental health status during hospitalization.

**Table 1:** Demographic characteristics of the participants.

Variable	*N*	%
*Gender*		
Male	563	43.4
Female	734	56.6
*Age (years)*		
≤ 60	683	52.7
> 60	614	47.3
*Region*		
Urban	1,136	87.6
Rural areas	161	12.4
*Marital status*		
Unmarried/divorced /widowed	192	14.8
Married	1,105	85.2
*Income for 2020 (CNY)*		
< 60,000	805	62.1
≥ 60,000	492	37.9
*Dwelling state*		
Living alone	158	12.2
Living with others	1,139	87.8
*Education level*		
Senior high school or below	921	71
Above senior high school	376	29
*Frequency of alcohol use per week*		
< 2	1,180	91
≥ 2	117	9
*Current smoker*		
No	1,136	87.6
Yes	161	12.4
*Clinical classification of COVID-19 patients*		
Asymptomatic	60	4.6
Mild	927	71.5
Moderate	132	10.2
Critically severe	178	13.7
*Experience at ICU*		
No	1,250	96.4
Yes	47	3.6
*Length of hospital stay (days)*		
≤ 20	686	52.9
> 20	611	47.1
*Complication*		
No	530	40.9
Yes	767	59.1
*Having received psychological or emotional counseling during hospitalization*		
No	1,225	94.4
Yes	72	5.6
*Perceived mental health status during hospitalization*		
Poor	314	24.2
Moderate	247	19
Good	736	56.7
*Having received psychological or emotional counseling before infection*		
No	1,169	90.1
Yes	128	9.9

### 3.2. Stigma and related psychological factors

The 12-item CSS-S’s total scores range from 12 to 48 with higher scores indicating a more stigmatizing attitude. The mean score in this study was 28.04 (SD = 7.33). The mean scores of fatigue, peace of mind, resilience, and social support were 6.38 (SD = 4.04), 24.70 (SD = 5.99), 56.82 (SD = 14.04), 14.25 (SD = 5.18), respectively. The prevalence of PTSD, anxiety, depression, and sleep disorder were 16.5, 28.8, 37.9, and 47.1%, respectively (Table 2).

**Table 2:** Descriptive statistics for CSS-S, FS-14, PoM, RSQ, social support, IES-R, GAD-7, PHQ-9 and PSQI.

Variable	*n*	%	Mean (SD)	Range
*Stigma (CSS-S)*				
Total score			28.04(7.33)	12–48
*Fatigue (FS-14)*				
Total score			6.38(4.04)	0–14
*Peace of mind (PoM)*				
Total score			24.70(5.99)	7–35
*Resilience (RSQ)*				
Total score			56.82(14.04)	16–80
*Social support*				
Total score			14.25(5.18)	0–20
*Post-traumatic stress disorder (IES-R)*				
No	1,083	83.5		
Yes	214	16.5		
*Anxiety (GAD-7)*				
No	923	71.2		
Yes	374	28.8		
*Depression (PHQ-9)*				
No	805	62.1		
Yes	492	37.9		
*Sleep disorder (PSQI)*				
No	686	52.9		
Yes	611	47.1		

CSS-S: COVID-19 Stigma Scale; FS-14: Fatigue Scale-14; PoM: Peace of Mind Scale; RSQ: Resilience Style Questionnaire; IES-R: Impact of Events Scale-Revised; GAD-7: Generalized Anxiety Disorder Questionnaire; PHQ-9: Patient Health Questionnaire; PSQI: Pittsburgh Sleep Quality Index.

### 3.3. Latent profile analysis

**Figure 1:**
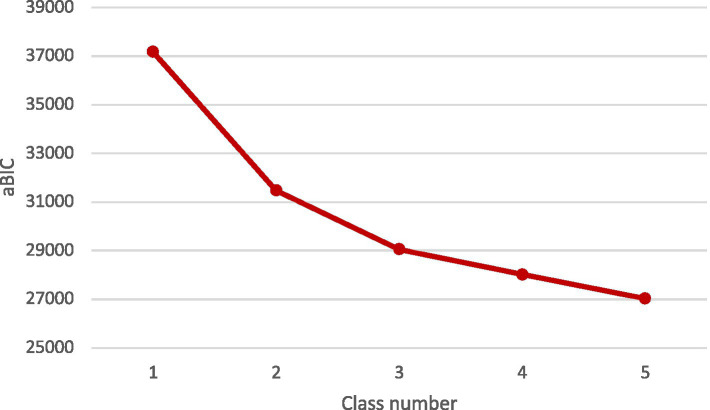
Scree plot of change trend of adjusted Bayesian Information Criterion (aBIC).

**Figure 2:**
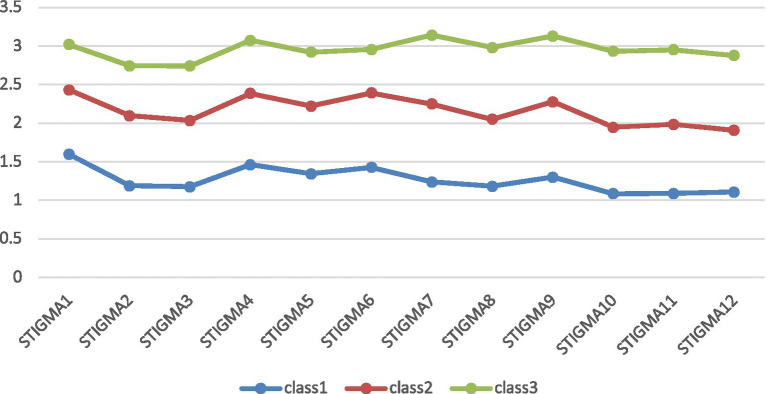
Three classes of the best-fitting 3-class model based on COVID-19 Stigma Scale (CSS-S).

Latent profile models (LPA) with one-to-five-class solutions were specified, and the fit indices of the 5 models are displayed in Table 3. The entropies of all classifications were above 0.9. The LMR and BLRT test were all statistically significant. The AIC, BIC and aBIC decreased with the increase of class number, and the scree plot of aBIC flattened out after the 3-class model (see Figure 1). Taken together, considering the model fit, parsimoniousness, and interpretability of the classes, the 3-class model was selected as the optimal model for the current sample, the distribution and conditional means of items of CSS-S on each class in the 3-class model are illustrated in Figure 2 and Table 4. In the 3-class model, the average latent class probabilities for most likely latent class membership (0.978, 0.977, and 0.972) demonstrate reasonable classification and good distinction (see Table 5). Given the conditional means of items on each class, we define Class1 (*n* = 166, 12.8%) as “low perceived COVID-19 stigma” group, Class2 (*n* = 663, 51.1%) as “moderate perceived COVID-19 stigma” group, and Class3 (*n* = 468, 36.1%) as “severe perceived COVID-19 stigma.”

**Table 3:** Model fit indices for latent profile models with different classes.

Class number	AIC	BIC	aBIC	Entropy	LMR	BLRT	Class membership probability
1	37121.342	37245.37	37169.134				1
2	31403.104	31594.313	31476.782	0.928	<0.001	<0.001	0.510/0.490
3	28949.821	29208.211	29049.386	0.944	<0.001	<0.001	0.128/0.361/0.511
4	27896.009	28221.581	28021.461	0.954	0.007	<0.001	0.125/0.369/0.469/0.037
5	26872.931	27265.684	27024.269	0.954	0.006	<0.001	0.137/0.360/0.307/0.160/0.036

**Table 4:** Conditional means of items of CSS-S on each class.

	Class1	Class2	Class3
STIGMA1	1.597	2.433	3.022
STIGMA2	1.188	2.097	2.744
STIGMA3	1.174	2.035	2.742
STIGMA4	1.463	2.387	3.074
STIGMA5	1.343	2.222	2.921
STIGMA6	1.428	2.394	2.956
STIGMA7	1.239	2.249	3.144
STIGMA8	1.183	2.05	2.981
STIGMA9	1.299	2.277	3.129
STIGMA10	1.084	1.948	2.934
STIGMA11	1.087	1.986	2.952
STIGMA12	1.108	1.907	2.877
Class membership probability	0.128	0.511	0.361

**Table 5:** Average latent class probabilities for most likely latent class membership by latent class.

Latent class	Latent class membership		
	1 (166)	2 (663)	3 (468)
1	0.978	0.022	0.000
2	0.008	0.972	0.021
3	0.000	0.023	0.977

### 3.4. Influencing factors of perceived COVID-19 stigma of RD

The result of univariate analysis showed that female (*χ*^2^ = 21.999, *p* < 0.001), older age (*χ*^2^ = 45.595, *p* < 0.001), being married (*χ*^2^ = 4.401, *p* = 0.111), low family income (*χ*^2^ = 23.261, *p* < 0.001), living with other people (*χ*^2^ = 7.456, *p* = 0.024), low education level (*χ*^2^ = 61.653, *p* < 0.001), having complication (*χ*^2^ = 10.117, *p* = 0.006), perceiving worse mental health status during hospitalization (*χ*^2^ = 48.489, *p* < 0.001), PTSD (*χ*^2^ = 73.360, *p* < 0.001), anxiety (*χ*^2^ = 74.878, *p* < 0.001), depression (*χ*^2^ = 70.081, *p* < 0.001), sleep disorder (*χ*^2^ = 70.875, *p* < 0.001), and fatigue (*F* = 21.220, *p* < 0.001) were positively associated with perceived COVID-19 stigma, while resilience (*F* = 22.030, *p* < 0.001), social support (*F* = 25.070, *p* < 0.001), and peace of mind (*F* = 39.130, *p* < 0.001) were negatively associated with perceived COVID-19 stigma among RD (see Table 6). These variables were further employed in stepwise multinomial logistic regression analysis with the “low perceived COVID-19 stigma” group as a reference. The result of stepwise multinomial logistic regression analysis showed that older age (AOR = 1.753, *p* = 0.004), living with other people (AOR = 2.152, *p* = 0.003), anxiety (AOR = 2.444, *p* = 0.004), and sleep disorder (AOR = 1.921, *p* = 0.002) were positively associated with moderate perceived COVID-19 stigma, while higher educational level (AOR = 0.624, *p* = 0.012) was negatively associated with moderate perceived COVID-19 stigma; Female (AOR = 1.674, *p* = 0.011), older age (AOR = 3.046, *p* < 0.001), living with other people (AOR = 2.037, *p* = 0.011), anxiety (AOR = 2.813, *p* = 0.001), and sleep disorder (AOR = 2.628, *p* < 0.001) were positively associated with severe perceived COVID-19 stigma, while higher educational level (AOR = 0.340, *p* < 0.001), social support (AOR = 0.953, *p* = 0.021), and peace of mind (AOR = 0.951, *p* = 0.008) were negatively associated with severe perceived COVID-19 stigma among RD (Table 7).

**Table 6:** Univariate analysis of influencing factors of perceived COVID-19 stigma of RD.

Variable	Classification of perceived stigma			*χ*^2^/*F*	*p*
	Low perceived COVID-19 stigma	Moderate perceived COVID-19 stigma	Severe perceived COVID-19 stigma		
Gender				21.999	<0.001
Male	86	313	164		
Female	80	350	304		
Age(years)				45.595	<0.001
≤ 60	116	372	195		
> 60	50	291	273		
Region				1.695	0.428
Urban	147	573	416		
Rural areas	19	90	52		
Marital status				4.401	0.111
Unmarried/divorced/widowed	33	89	70		
Married	133	574	398		
Family income for 2020 (CNY)				23.261	<0.0001
< 60,000	83	396	326		
≥ 60,000	83	267	142		
Dwelling state				7.456	0.024
Living alone	28	66	64		
Living with other people	138	597	404		
Education level				61.653	<0.001
Senior high school or below	88	446	387		
Above senior high school	78	217	81		
Frequency of alcohol use per week				1.197	0.5496
< 2	149	600	431		
≥ 2	17	63	37		
Current smoker				2.349	0.309
No	140	580	416		
Yes	26	83	52		
Clinical classification of COVID-19 patients				7.743	0.2575
Asymptomatic	8	36	16		
Mild	130	464	333		
Moderate	12	69	51		
Critical severe	16	94	68		
Experience at ICU				0.956	0.6202
No	162	639	449		
Yes	4	24	19		
Length of hospital stay(days)				0.937	0.6259
≤ 20	83	358	245		
> 20	83	305	223		
Complication				10.117	0.006
No	86	267	177		
Yes	80	396	291		
Having received psychological or emotional counseling during hospitalization				0.604	0.7396
No	157	629	439		
Yes	9	34	29		
Perceived mental health status during hospitalization				48.489	<0.001
Poor	22	129	163		
Moderate	39	129	79		
Good	105	405	226		
Having received psychological or emotional counseling before infection				0.030	0.9852
No	149	598	422		
Yes	17	65	46		
PTSD				73.360	<0.001
No	157	589	337		
Yes	9	74	131		
Anxiety				74.878	<0.001
No	150	501	272		
Yes	16	162	196		
Depression				70.081	<0.001
No	138	438	229		
Yes	28	225	239		
Sleep disorder				70.875	<0.001
No	125	376	185		
Yes	41	287	283		
Fatigue	5.193 ± 4.137	6.050 ± 3.931	7.263 ± 3.982	21.220	<0.001
Resilience	58.693 ± 19.189	58.750 ± 12.896	53.436 ± 12.772	22.030	<0.001
Social support	15.223 ± 5.401	14.944 ± 4.761	12.928 ± 5.399	25.070	<0.001
Peace of mind	26.530 ± 6.807	25.558 ± 5.698	22.846 ± 5.614	39.130	<0.001

**Table 7:** Multinomial logistic regression analysis of influencing factors of perceived COVID-19 stigma of RD.

Variable	Moderate perceived stigma				Severe perceived stigma			
	AOR	95%CI		*p*	AOR	95%CI		*p*
		LL	UL			LL	UL	
Gender								
Male	1				1			
Female	1.063	0.744	1.519	0.736	1.674	1.128	2.483	0.011
Age(years)								
≤ 60	1				1			
> 60	1.753	1.192	2.577	0.004	3.046	2.009	4.618	<0.001
Dwelling state								
Living alone	1				1			
Living with other people	2.152	1.304	3.553	0.003	2.037	1.181	3.515	0.011
Education level								
Senior high school or below	1				1			
Above senior high school	0.624	0.432	0.903	0.012	0.340	0.221	0.522	<0.001
PTSD								
No	1				1			
Yes	1.082	0.495	2.367	0.844	2.014	0.915	4.431	0.082
Anxiety								
No	1				1			
Yes	2.444	1.327	4.501	0.004	2.813	1.499	5.276	0.001
Sleep disorder								
No	1				1			
Yes	1.921	1.275	2.895	0.002	2.628	1.695	4.072	<0.001
Social support	1.005	0.966	1.046	0.803	0.953	0.914	0.993	0.021
Peace of mind	0.998	0.965	1.032	0.891	0.951	0.917	0.987	0.008

LL: lower limit; UL: upper limit.

### 3.5. Receiver operating characteristic analysis

**Figure 3:**
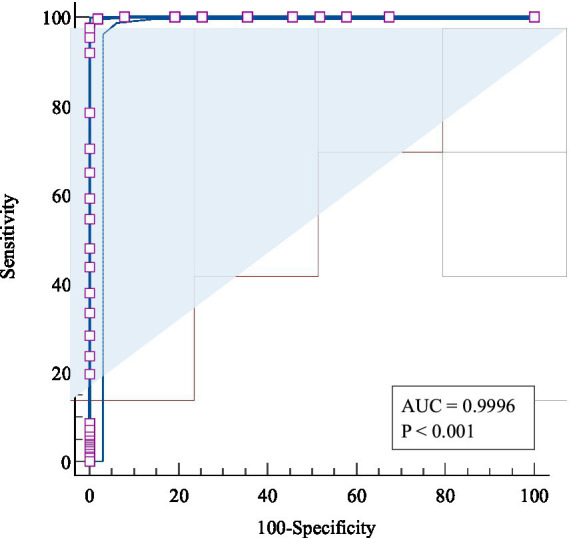
The receiver operating characteristic (ROC) curve of the CSS-S for screening perceived COVID-19 stigma.

To identify the optimal cut-off value of CSS-S for screening perceived COVID-19 stigma among RD, participants assigned to the “low perceived COVID-19 stigma” group in LPA were defined as “non-cases” (i.e., no stigma), and those assigned in “moderate perceived COVID-19 stigma” and “severe perceived COVID-19 stigma” groups were defined as “cases” (i.e., probable stigma). The ROC curve was then plotted for the total score of CSS-S using the binary outcome, with an AUC value of 99.96% (*p* < 0.001), indicating a good predictive capacity for perceived COVID-19 stigma (see Figure 3). The diagnostic criteria and indices are illustrated in Table 8. The optimal cut-off value was ≥ 20, where the sensitivity, specificity, and Youden’s index value were 0.996, 0.982, and 0.978, respectively.

**Table 8:** Criterion values and coordinates of ROC curve for perceived COVID-19 stigma.

Criterion	Sensitivity	Specificity	Youden’s index
≥ 12	1.000	0.000	0.000
> 19	1.000	0.922	0.922
> 20	0.996	0.982	0.978
> 21	0.975	1.000	0.975
> 48	0.000	1.000	0.000

## 4. Discussion

The cross-sectional study employs LPA to assess the characteristics of perceived COVID-19 stigma among RD and analyzes its psycho-socio contributing factors. Perceived stigma of RD was divided into three categories in this study. We measured the demographic characteristics and some possible psychological predictors of perceived COVID-19 stigma. Generally, older age, living with other people, anxiety, and sleep disorder were positively associated with moderate perceived COVID-19 stigma, while higher educational level was negatively associated with moderate perceived COVID-19 stigma; female, older age, living with other people, anxiety, and sleep disorder were positively associated with severe perceived COVID-19 stigma, while higher educational level, social support, and peace of mind were negatively associated with severe perceived COVID-19 stigma among RD. The cut-off point of the stigma scale was determined at 20 using ROC analysis.

This study classified COVID-19 RD into three groups according to the stigma level: “low perceived COVID-19 stigma,” “moderate perceived COVID-19 stigma,” and “severe perceived COVID-19 stigma” group. Only 12.8% of RD were categorized into the “low perceived COVID-19 stigma” group, which indicated the lowest levels of stigma and reported the lowest level of psychological risk factors. The majority belonged to the “moderate perceived COVID-19 stigma” (51.1%).

Compared with the “low perceived COVID-19 stigma” group, anxiety and sleep disorder were positively associated with moderate perceived stigma. Similar to previously published studies, anxiety was a major risk factor for stigma. In a study that evaluated the depression and anxiety symptoms among 174 patients who recovered from symptomatic COVID-19 infection in Saudi Arabia, the stigma scores were significantly associated with higher scores on anxiety (63). Some other studies on people living with epilepsy, dementia, and cancer patients also demonstrated that anxiety is one of the psychosocial determinants of perceived stigma (64–66). Therefore, mitigating the anxiety symptoms is essential to decrease the stigma among RD. Emotional regulation, mindfulness, and experiential techniques are possible solutions to improve social anxiety disorder symptoms (67). RD could also try exercise, yoga, and meditation, which were proven to have modest positive effect on assisting their anxiety alleviation (68). Hospitals and communities should assess the anxiety level of COVID-19 RD to detect anxiety as early as possible. For RD with anxiety symptoms, the community should provide them with knowledge and stress coping strategies, and provide training to help them manage emotions. Psychological interventions like mindfulness-based therapy could be implemented by government or community to alleviate anxiety symptoms (69). The society should be less hostile to RD. It is necessary for social media to refute false information, strengthen the information guidance of social media, and output positive information, so as to avoid the anxiety mood in origin.

Our study also found that sleep disorders is a determinant of moderate perceived stigma in RD. Previous studies showed that 29.5% of the COVID-19 hospitalized patients had sleep disorders (70). Poor sleep quality was associated with stigma (71). Cognitive behavior therapy is aimed at treating insomnia by avoiding behaviors and thoughts that might develop into sleep disorders (72). RD with sleep disorders could use this method on their own to improve their sleep quality. Effective programs based on the therapy could also be embedded in smartphones to assist their sleep promotion process (73). In addition, progressive muscular relaxation is an effective way to help COVID-19 patients feel less anxious and have better quality sleep (74).

The “severe perceived COVID-19 stigma” group reported three more risk factors compared with “moderate perceived COVID-19 stigma” group, including female gender, insufficient social support and peace of mind. Female gender is a risk factor of “long-covid” syndrome and tend to have a higher proportion of physical and psychological symptoms than male (75). Because of the more severe illness and torment they suffered, they might find it difficult to maintain a good mentality toward the stigmatized attitudes. A low perceived level of social support prevailed during the pandemic due to the shutdown of many places, like schools, markets, and workplaces to avoid transmission of the virus (76). RD facing such conditions may arouse a sense of isolation and vulnerability, which would cause severe stigma. Perceived social support and use of adaptive coping strategies were found to affect individuals’ psychological adjustment and resilience (77). Interventions like in-person interview, supportive psychotherapy, and positive attention would improve their social support and could be considered widely promoted (78). Peace of mind might increase one’s self awareness and attitude toward the surroundings, and indirectly reduce the sense of being stigmatized. A previous study on female patients with schizophrenia also identified that enhancing peace of mind will help reduce stigma level (79).

Our study determined 20 as the cut-off score for CSS-S by LPA and ROC analysis, which may guide future epidemiological studies on COVID-19 stigma. The cut-off value is instructive for clinical practice in COVID-19 RD mental health promotion. Hospitals are suggested to collect stigma information of discharged patients and carry out relevant psychological intervention for patients whose scores exceed 20.

Although our team have analyzed the same population in advance and explored the prevalence and influencing factors of anxiety and depression in RD (80), a further analysis in this study provided insightful observations from a different perspective. This study enriched our knowledge on the association between mental health and perceived stigma among RD, and provided possible suggestions for the authorities and the society to reduce perceived COVID-19 stigma in the future. However, it has several limitations. First, this cross-sectional study has its inherent limitations, for it contains no dimension of time to support a causal relationship. Second, the study was conducted more than 18 months after the COVID-19 patients were discharged, which may cause recall bias. Third, convenience sampling may decrease the representativeness of the population. Fourth, stigma contains two factors, namely “public stigma” and “self-perceived stigma.” In this study, we only mention the latter. Further studies should measure stigma more comprehensively in a representative sample.

## 5. Conclusion

This study provides an insightful result of the prevalence and influencing factors of perceived stigma among RD in Wuhan. Stigma among COVID-19 RD could be divided into 3 groups: “low perceived COVID-19 stigma,” “moderate perceived COVID-19 stigma,” and “severe perceived COVID-19 stigma” group. Based on the cut-off value we explored, the high proportion of perceived stigma level highlights the importance of solving the stigma and discrimination problem, for its impact on personal and community well-being. Therefore, it is essential to mitigate the psychological problems and reduce the perceived stigma level of RD as part of the response toward the COVID-19 pandemic. Psychological interventions on anxiety, sleep disorder, and social support are suggested to alleviate mental health problems and stigma among this population. Additionally, this study discovered the precise cut-off value for CSS-S, which provides a valuable tool for screening perceived stigma among future COVID-19 patients and can be used to identify the patients in noosed of tailored interventions.

## Data availability statement

The raw data supporting the conclusions of this article will be made available by the authors, without undue reservation.

## Ethics statement

The studies involving human participants were reviewed and approved by the Ethics Review Board of the Institute of Pathogen Biology, Chinese Academy of Medical Sciences (IPB-2020-22), and the Research Ethics Committee of the hospital (2021001, 2021028). The patients/participants provided their written informed consent to participate in this study.

## Author contributions

ZD and YW prepared the first draft and analyzed the data. XS provided overall guidance and managed the overall project. WX, HW, YH, MS, JF, XC, MJ, ZL, DC, and WM were responsible for the questionnaire survey and data management. YW, ZD, and XS prepared and finalized the manuscript on the basis of comments from other authors. All authors contributed to the article and approved the submitted version.

## Funding

This research was funded by the Innovative Engineering Program sponsored by Chinese Academy of Medical Sciences (2020-I2M-2-015).

## Conflict of interest

The authors declare that the research was conducted in the absence of any commercial or financial relationships that could be construed as a potential conflict of interest.

## Publisher’s note

All claims expressed in this article are solely those of the authors and do not necessarily represent those of their affiliated organizations, or those of the publisher, the editors and the reviewers. Any product that may be evaluated in this article, or claim that may be made by its manufacturer, is not guaranteed or endorsed by the publisher.

## References

[1] Sohrabi C Alsafi Z Oneill N Khan M Kerwan A Al-Jabir A. World Health Organization declares global emergency: a review of the 2019 novel coronavirus (COVID-19). Int J Surg. (2020) 76:71–6. doi: 10.1016/j.ijsu.2020.02.034, PMID: 32112977 PMC7105032

[2] Saladino V Algeri D Auriemma V. The psychological and social impact of Covid-19: new perspectives of well-being. Front Psychol. (2020) 11:577684. doi: 10.3389/fpsyg.2020.577684, PMID: 33132986 PMC7561673

[3] Uvais NA Aziz F Hafeeq B. COVID-19-related stigma and perceived stress among dialysis staff. J Nephrol. (2020) 33:1121–2. doi: 10.1007/s40620-020-00833-x, PMID: 32804354 PMC7429935

[4] Liu CH Chen YJ Chen JS Fan CW Hsieh MT Lin CY. Burdens on caregivers of patients with stroke during a pandemic: relationships with support satisfaction, psychological distress, and fear of COVID-19. BMC Geriatr. (2022) 22:958. doi: 10.1186/s12877-022-03675-3, PMID: 36514006 PMC9745281

[5] Dong F Liu H-l Dai N Yang M Liu J-p. A living systematic review of the psychological problems in people suffering from COVID-19. J Affect Disord. (2021) 292:172–88. doi: 10.1016/j.jad.2021.05.060, PMID: 34126309 PMC8169237

[6] Sangma RD Kumar P Nerli LM Khanna AM Vasavada DA Tiwari DS. Social stigma and discrimination in coronavirus Disease-2019 survivors and its changing trend: a longitudinal study at tertiary care center Gujarat, India. Asian J Soc Health Behav. (2022) 5:68. doi: 10.4103/shb.shb_12_22

[7] Patel BR Khanpara BG Mehta PI Patel KD Marvania NP. Evaluation of perceived social stigma and burnout, among health-care workers working in covid-19 designated hospital of India: a cross-sectional study. Asian J Soc Health Behav. (2021) 4:156. doi: 10.4103/shb.shb_54_21

[8] Torjesen I. Covid-19 patients discharged from hospital have "substantially higher risk" of adverse outcomes and need monitoring. BMJ. (2022) 376:o265. doi: 10.1136/bmj.o265 35105538

[9] Xiong LJ Zhong BL Cao XJ Xiong HG Huang M Ding J. Possible posttraumatic stress disorder in Chinese frontline healthcare workers who survived COVID-19 6 months after the COVID-19 outbreak: prevalence, correlates, and symptoms. Transl Psychiatry. (2021) 11:374. doi: 10.1038/s41398-021-01503-7, PMID: 34226510 PMC8256400

[10] Yuan K Gong Y-M Liu L Sun Y-K Tian S-S Wang Y-J. Prevalence of posttraumatic stress disorder after infectious disease pandemics in the twenty-first century, including COVID-19: a meta-analysis and systematic review. Mol Psychiatry. (2021) 26:4982–98. doi: 10.1038/s41380-021-01036-x, PMID: 33542468 PMC7861006

[11] Li L Wu MS Tao J Wang W He J Liu R. A follow-up investigation of mental health among discharged COVID-19 patients in Wuhan. China Front Public Health. (2021) 9:640352. doi: 10.3389/fpubh.2021.640352, PMID: 33912531 PMC8071993

[12] Guido CA Lucidi F Midulla F Zicari AM Bove E Avenoso F. Neurological and psychological effects of long COVID in a young population: a cross-sectional study. Front Neurol. (2022) 13:925144. doi: 10.3389/fneur.2022.925144, PMID: 36062008 PMC9428748

[13] van Kessel SAM Olde Hartman TC Lucassen PLBJ van Jaarsveld CHM. Post-acute and long-COVID-19 symptoms in patients with mild diseases: a systematic review. Fam Pract. (2021) 39:159–67. doi: 10.1093/fampra/cmab076 PMC8414057 34268556

[14] Rogers JP Chesney E Oliver D Pollak TA McGuire P Fusar-Poli P. Psychiatric and neuropsychiatric presentations associated with severe coronavirus infections: a systematic review and meta-analysis with comparison to the COVID-19 pandemic. Lancet Psychiatry. (2020) 7:611–27. doi: 10.1016/S2215-0366(20)30203-0, PMID: 32437679 PMC7234781

[15] Saiz J Munoz M Ausin B Gonzalez-Sanguino C Angel Castellanos M Vaquero C. Effects of COVID-19 lockdown on perceived discrimination and internalized stigma in people with previous mental disorder diagnoses in Spain. Am J Orthopsychiatry. (2021) 91:407–11. doi: 10.1037/ort0000542, PMID: 34138629

[16] Mickelson KD. Perceived stigma, social support, and depression. Personal Soc Psychol Bull. (2001) 27:1046–56. doi: 10.1177/0146167201278011

[17] Xiao S Luo D Xiao Y. Survivors of COVID-19 are at high risk of posttraumatic stress disorder. Glob Health Res Policy. (2020) 5:29. doi: 10.1186/s41256-020-00155-2, PMID: 32514428 PMC7273810

[18] Kaseda ET Levine AJ. Post-traumatic stress disorder: a differential diagnostic consideration for COVID-19 survivors. Clin Neuropsychol. (2020) 34:1498–514. doi: 10.1080/13854046.2020.1811894, PMID: 32847484

[19] Lu MY Ahorsu DK Kukreti S Strong C Lin YH Kuo YJ. The prevalence of post-traumatic stress disorder symptoms, sleep problems, and psychological distress among COVID-19 frontline healthcare Workers in Taiwan. Front Psych. (2021) 12:705657. doi: 10.3389/fpsyt.2021.705657, PMID: 34322044 PMC8312888

[20] Schou TM Joca S Wegener G Bay-Richter C. Psychiatric and neuropsychiatric sequelae of COVID-19—a systematic review. Brain Behav Immun. (2021) 97:328–48. doi: 10.1016/j.bbi.2021.07.018, PMID: 34339806 PMC8363196

[21] Chi P Li X Zhao J Zhao G. Vicious circle of perceived stigma, enacted stigma and depressive symptoms among children affected by HIV/AIDS in China. AIDS Behav. (2014) 18:1054–62. doi: 10.1007/s10461-013-0649-z, PMID: 24158487 PMC4000575

[22] Hao F Tam W Hu X Tan W Jiang L Jiang X. A quantitative and qualitative study on the neuropsychiatric sequelae of acutely ill COVID-19 inpatients in isolation facilities. Transl Psychiatry. (2020) 10:355. doi: 10.1038/s41398-020-01039-2, PMID: 33077738 PMC7570419

[23] Conversano C Di Giuseppe M Miccoli M Ciacchini R Gemignani A Orrù G. Mindfulness, age and gender as protective factors against psychological distress during COVID-19 pandemic. Front Psychol. (2020) 11:1900. doi: 10.3389/fpsyg.2020.01900, PMID: 33013503 PMC7516078

[24] PeConga EK Gauthier GM Holloway A Walker RSW Rosencrans PL Zoellner LA. Resilience is spreading: mental health within the COVID-19 pandemic. Psychol Trauma. (2020) 12:S47–8. doi: 10.1037/tra0000874, PMID: 32496106 PMC7398149

[25] Chiu H-C Lin C-Y Kuo Y-L Hou W-L Shu B-C. Resilience among women with breast cancer surviving longer than five years: the relationship with illness perception and body image. Eur J Oncol Nurs. (2022) 62:102254 doi: 10.1016/j.ejon.2022.102254 36621263

[26] Lin YH Chen JS Huang PC Lu MY Strong C Lin CY. Factors associated with insomnia and suicidal thoughts among outpatients, healthcare workers, and the general population in Taiwan during COVID-19 pandemic: a cross-sectional study. BMC Public Health. (2022) 22:2135. doi: 10.1186/s12889-022-14557-z, PMID: 36411400 PMC9676880

[27] Olashore AA Akanni OO Fela-Thomas AL Khutsafalo K. The psychological impact of COVID-19 on health-care workers in African countries: a systematic review. Asian J. Soc. Health Behav. (2021) 4:85. doi: 10.4103/shb.shb_32_21

[28] Wu YP Ahorsu DK Chen JS Lee CH Lin CY Griffiths MD. The role of demographic factors, mindfulness and perceived stress in resilience among nurses: a cross sectional study. J Nurs Manag. (2022) 30:3093–101. doi: 10.1111/jonm.13715, PMID: 35695219

[29] Rashnuodi P Nourollahi-Darabad M Afshari D Shirali GA Amiri A Rotkhali E. The effect of resilience indicators on the job stress level among nurses: a predictor study. Asian J Soc Health Behav. (2022) 5:138. doi: 10.4103/shb.shb_4_22

[30] Kadian S Joseph J Pal S Devi R. Brief resilience interventions for mental health among college students: randomized controlled trial. Asian J Soc Health Behav. (2022) 5:131. doi: 10.4103/shb.shb_28_22

[31] Yadav AK Mangal V Devarakonda R Srivastava K. Perceived stigma among the patients of coronavirus disease-19 admitted at a dedicated COVID-19 hospital in northern India: a cross-sectional study. Ind Psychiatry J. (2021) 30:118–22. doi: 10.4103/ipj.ipj_13_21, PMID: 34483535 PMC8395555

[32] Corrigan PW Rao D. On the self-stigma of mental illness: stages, disclosure, and strategies for change. Can J Psychiatr. (2012) 57:464–9. doi: 10.1177/070674371205700804, PMID: 22854028 PMC3610943

[33] Imran N Afzal H Aamer I Hashmi A Shabbir B Asif A. Scarlett letter: a study based on experience of stigma by COVID-19 patients in quarantine. Pak J Med Sci. (2020) 36:1471–7. doi: 10.12669/pjms.36.7.3606, PMID: 33235559 PMC7674879

[34] Bronfenbrenner U. The Ecology of Human Development: Experiments by Nature and Design. Cambridge, MA: Harvard University Press (1979).

[35] Horowitz M Wilner N Alvarez W. Impact of event scale: a measure of subjective stress. Psychosom Med. (1979) 41:209–18. doi: 10.1097/00006842-197905000-00004, PMID: 472086

[36] Weiss DS Marmar CR. The Impact of Event Scale—Revised. Assessing Psychological Trauma and PTSD. New York, NY: The Guilford Press; (1997). p. 399–411.

[37] Zheng Y Xiao L Huang Y Wang Q Xie Y Wang H. Possible vicarious traumatization among psychiatric inpatients during the remission phase of the COVID-19: a single-center cross-sectional study. Front Psych. (2021) 12:677082. doi: 10.3389/fpsyt.2021.677082, PMID: 34504442 PMC8421644

[38] Spitzer RL Kroenke K Williams JB Lowe B. A brief measure for assessing generalized anxiety disorder: the GAD-7. Arch Intern Med. (2006) 166:1092–7. doi: 10.1001/archinte.166.10.1092 16717171

[39] Miao Q Xie L Xing B Wang X Tang S Luo H. Emotional states and coping methods in nursing and non-nursing students responding to COVID-19: a cross-sectional study in China. BMJ Open. (2021) 11:e054007. doi: 10.1136/bmjopen-2021-054007, PMID: 34446505 PMC8392730

[40] Gong J Chen G Qi Z Zhong S Su T Pan Y. Psychological effects of people isolated in Hubei due to COVID-19 epidemic. Front Psych. (2021) 12:597894. doi: 10.3389/fpsyt.2021.597894, PMID: 34393837 PMC8355422

[41] Kroenke K Spitzer RL Williams JB. The PHQ-9: validity of a brief depression severity measure. J Gen Intern Med. (2001) 16:606–13. doi: 10.1046/j.1525-1497.2001.016009606.x, PMID: 11556941 PMC1495268

[42] Yao YY Wei ZJ Zhang YC Li X Gong L Zhou JW. Functional disability after ischemic stroke: a community-based cross-sectional study in Shanghai. China Front Neurol. (2021) 12:649088. doi: 10.3389/fneur.2021.649088, PMID: 34512499 PMC8427524

[43] Hou T Xie Y Mao X Liu Y Zhang J Wen J. The mediating role of loneliness between social support and depressive symptoms among Chinese rural adolescents during COVID-19 outbreak: a comparative study between left-behind and non-left-behind students. Front Psych. (2021) 12:740094. doi: 10.3389/fpsyt.2021.740094, PMID: 34497549 PMC8420998

[44] Buysse DJ Reynolds CF 3rd Monk TH Berman SR Kupfer DJ. The Pittsburgh sleep quality index: a new instrument for psychiatric practice and research. Psychiatry Res. (1989) 28:193–213. doi: 10.1016/0165-1781(89)90047-4, PMID: 2748771

[45] Carpenter JS Andrykowski MA. Psychometric evaluation of the Pittsburgh sleep quality index. J Psychosom Res. (1998) 45:5–13. doi: 10.1016/S0022-3999(97)00298-5 9720850

[46] Liu X Tang M Hu M Wang A Wu H Zhao G. The validity and reliability of Pittsburgh sleep quality index. Chin J Psychiatry. (1996) 29:103–7.

[47] Chalder T Berelowitz G Pawlikowska T Watts L Wessely S Wright D. Development of a fatigue scale. J Psychosom Res. (1993) 37:147–53. doi: 10.1016/0022-3999(93)90081-P 8463991

[48] Jing MJ Lin WQ Wang Q Wang JJ Tang J Jiang ES. Reliability and construct validity of two versions of Chalder fatigue scale among the general population in mainland China. Int J Environ Res Public Health. (2016) 13:19. doi: 10.3390/ijerph13010147, PMID: 26805863 PMC4730538

[49] Mak WWS Ng ISW Wong CCY Law RW. Resilience style questionnaire: development and validation among college students and cardiac patients in Hong Kong. Assessment. (2019) 26:706–25. doi: 10.1177/1073191116683798, PMID: 28006974

[50] Li J Chen YP Zhang J Lv MM Valimaki M Li YF. The mediating role of resilience and self-esteem between life events and coping styles among rural left-behind adolescents in China: a cross-sectional study. Front Psych. (2020) 11:560556. doi: 10.3389/fpsyt.2020.560556, PMID: 33329099 PMC7714763

[51] Lin J Ren YH Gan HJ Chen Y Huang YF You XM. Factors associated with resilience among non-local medical workers sent to Wuhan, China during the COVID-19 outbreak. BMC Psychiatry. (2020) 20:417. doi: 10.1186/s12888-020-02821-8, PMID: 32831045 PMC7443813

[52] Li J Mo PK Wu AM Lau JT. Roles of self-stigma, social support, and positive and negative affects as determinants of depressive symptoms among HIV infected men who have sex with men in China. AIDS Behav. (2017) 21:261–73. doi: 10.1007/s10461-016-1321-1, PMID: 26896120 PMC4992470

[53] Lee Y-C Lin Y-C Huang C-L Fredrickson BL. The construct and measurement of peace of mind. J Happiness Stud. (2013) 14:571–90. doi: 10.1007/s10902-012-9343-5

[54] Li JB Wu AMS Feng LF Deng Y Li JH Chen YX. Classification of probable online social networking addiction: a latent profile analysis from a large-scale survey among Chinese adolescents. J Behav Addict. (2020) 9:698–708. doi: 10.1556/2006.2020.00047, PMID: 32829311 PMC8943659

[55] van Smeden M Naaktgeboren CA Reitsma JB Moons KG de Groot JA. Latent class models in diagnostic studies when there is no reference standard--a systematic review. Am J Epidemiol. (2014) 179:423–31. doi: 10.1093/aje/kwt286, PMID: 24272278

[56] Fu H Si L Guo R. What is the optimal cut-off point of the 10-item Center for Epidemiologic Studies Depression Scale for screening depression among Chinese individuals aged 45 and over? An exploration using latent profile analysis. Front Psych. (2022) 13:820777. doi: 10.3389/fpsyt.2022.820777, PMID: 35360127 PMC8963942

[57] Berlin KS Williams NA Parra GR. An introduction to latent variable mixture modeling (part 1): overview and cross-sectional latent class and latent profile analyses. J Pediatr Psychol. (2014) 39:174–87. doi: 10.1093/jpepsy/jst084, PMID: 24277769

[58] Lubke GH Muthen B. Investigating population heterogeneity with factor mixture models. Psychol Methods. (2005) 10:21–39. doi: 10.1037/1082-989X.10.1.21 15810867

[59] Bondjers K Willebrand M Arnberg FK. Similarity in symptom patterns of posttraumatic stress among disaster-survivors: a three-step latent profile analysis. Eur J Psychotraumatol. (2018) 9:1546083. doi: 10.1080/20008198.2018.1546083, PMID: 30479702 PMC6249547

[60] Meehl PE Yonce LJ. Taxometric analysis: II. Detecting Taxonicity using covariance of two quantitative indicators in successive intervals of a third indicator (Maxcov procedure). Psychol Rep. (1996) 78:1091–227.

[61] Yu M Zhou H Wang M Tang X. The heterogeneity of social anxiety symptoms among Chinese adolescents: results of latent profile analysis. J Affect Disord. (2020) 274:935–42. doi: 10.1016/j.jad.2020.06.003, PMID: 32664035

[62] Magidson J Vermunt JK. Latent class models for clustering: A comparison with K-means. Canadian Journal of Marketing Research. (2002) 20:36–43.

[63] Alkathiri MA Almohammed OA Alqahtani F AlRuthia Y. Associations of depression and anxiety with stigma in a sample of patients in Saudi Arabia who recovered from COVID-19. Psychol Res Behav Manag. (2022) 15:381–90. doi: 10.2147/PRBM.S350931, PMID: 35237078 PMC8882661

[64] Shi Y Wang S Ying J Zhang M Liu P Zhang H. Correlates of perceived stigma for people living with epilepsy: a meta-analysis. Epilepsy Behav. (2017) 70:198–203. doi: 10.1016/j.yebeh.2017.02.022, PMID: 28431368

[65] Burgener SC Buckwalter K Perkhounkova Y Liu MF. The effects of perceived stigma on quality of life outcomes in persons with early-stage dementia: longitudinal findings: part 2. Dementia. (2015) 14:609–32. doi: 10.1177/1471301213504202, PMID: 24339117

[66] Else-Quest NM LoConte NK Schiller JH Hyde JS. Perceived stigma, self-blame, and adjustment among lung, breast and prostate cancer patients. Psychol Health. (2009) 24:949–64. doi: 10.1080/08870440802074664, PMID: 20205038

[67] Rodebaugh TL Holaway RM Heimberg RG. The treatment of social anxiety disorder. Clin Psychol Rev. (2004) 24:883–908. doi: 10.1016/j.cpr.2004.07.007 15501560

[68] Saeed SA Cunningham K Bloch RM. Depression and anxiety disorders: benefits of exercise, yoga, and meditation. Am Fam Physician. (2019) 99:620–7. 31083878

[69] Ho CS Chee CY Ho RC. Mental health strategies to combat the psychological impact of coronavirus disease 2019 (COVID-19) beyond paranoia and panic. Ann Acad Med Singap. (2020) 49:155–60. doi: 10.47102/annals-acadmedsg.202043, PMID: 32200399

[70] Kong X Zheng K Tang M Kong F Zhou J Diao L. Prevalence and factors associated with depression and anxiety of hospitalized patients with COVID-19. medRxiv. (2020). doi: 10.1101/2020.03.24.20043075 [Preprint]

[71] Fu L Wang B Chan PSF Luo D Zheng W Ju N. Associations between COVID-19 related stigma and sleep quality among COVID-19 survivors six months after hospital discharge. Sleep Med. (2022) 91:273–81. doi: 10.1016/j.sleep.2021.10.020, PMID: 34802891 PMC8529895

[72] Cunnington D Junge MF Fernando AT. Insomnia: prevalence, consequences and effective treatment. Med J Aust. (2013) 199:S36–40. doi: 10.5694/mja13.10718, PMID: 24138364

[73] Alimoradi Z Broström A Tsang HWH Griffiths MD Haghayegh S Ohayon MM. Sleep problems during COVID-19 pandemic and its’ association to psychological distress: a systematic review and meta-analysis. EClinicalMedicine. (2021) 36:100916. doi: 10.1016/j.eclinm.2021.100916, PMID: 34131640 PMC8192091

[74] Liu K Chen Y Wu D Lin R Wang Z Pan L. Effects of progressive muscle relaxation on anxiety and sleep quality in patients with COVID-19. Complement Ther Clin Pract. (2020) 39:101132. doi: 10.1016/j.ctcp.2020.101132, PMID: 32379667 PMC7102525

[75] Bai F Tomasoni D Falcinella C Barbanotti D Castoldi R Mulè G. Female gender is associated with long COVID syndrome: a prospective cohort study. Clin Microbiol Infect. (2022) 28:611.e9–611.e16. doi: 10.1016/j.cmi.2021.11.002 PMC8575536 34763058

[76] Lai J Ma S Wang Y Cai Z Hu J Wei N. Factors associated with mental health outcomes among health care workers exposed to coronavirus disease 2019. JAMA Netw Open. (2020) 3:e203976. doi: 10.1001/jamanetworkopen.2020.3976 32202646 PMC7090843

[77] Patil ST Datar MC Shetty JV Naphade NM. “Psychological consequences and coping strategies of patients undergoing treatment for COVID-19 at a tertiary care hospital”: a qualitative study. Asian J Soc Health Behav. (2021) 4:62. doi: 10.4103/shb.shb_5_21

[78] Yang X Yang X Kumar P Cao B Ma X Li T. Social support and clinical improvement in COVID-19 positive patients in China. Nurs Outlook. (2020) 68:830–7. doi: 10.1016/j.outlook.2020.08.008, PMID: 32980152 PMC7444976

[79] Tang Q Yang S Liu C Li L Chen X Wu F. Effects of mindfulness-based cognitive therapy on stigma in female patients with schizophrenia. Front Psych. (2021) 12:694575. doi: 10.3389/fpsyt.2021.694575, PMID: 34366925 PMC8342917

[80] Dai Z Xiao W Wang H Wu Y Huang Y Si M. Influencing factors of anxiety and depression of discharged COVID-19 patients in Wuhan, China. PLoS One. (2022) 17:e0276608. doi: 10.1371/journal.pone.0276608, PMID: 36383607 PMC9668158

